# The medium-term consequences of a COVID-19 lockdown on lifestyle among Spanish older people with hypertension, pulmonary disease, cardiovascular disease, musculoskeletal disease, depression, and cancer

**DOI:** 10.4178/epih.e2022026

**Published:** 2022-02-21

**Authors:** Irene Rodríguez-Gómez, Coral Sánchez-Martín, Francisco J. García-García, Esther García-Esquinas, Marta Miret, Germán Vicente-Rodriguez, Narcís Gusi, Asier Mañas, José A. Carnicero, Marcela Gonzalez-Gross, José L. Ayuso-Mateos, Fernando Rodríguez-Artalejo, Leocadio Rodríguez-Mañas, Ignacio Ara

**Affiliations:** 1 GENUD-Toledo Research Group, Universidad de Castilla-La Mancha, Toledo, Spain; 2 CIBER of Frailty and Healthy Aging (CIBERFES), Madrid, Spain; 3 Hospital Virgen del Valle, Complejo Hospitalario de Toledo, Toledo, Spain; 4 Department of Preventive Medicine and Public Health, School of Medicine, Universidad Autónoma de Madrid-IdiPaz and CIBERESP, Madrid, Spain; 5 IMDEA-Food Institute, CEI UAM+CSIC, Madrid, Spain; 6 Department of Psychiatry, School of Medicine, Universidad Autónoma de Madrid, Spain; 7 Instituto de Salud Carlos III, Centro de Investigación Biomédica en Red de Salud Mental (CIBERSAM), Madrid, Spain; 8 Department of Psychiatry, Hospital Universitario de La Princesa, IIS-Princesa, Madrid, Spain; 9 GENUD Research Group, FIMS Collaborating Center of Sports Medicine, Instituto Agroalimentario de Aragón -IA2-(CITA-Universidad de Zaragoza), Department of Physiatry and Nursing, University of Zaragoza, Spain, Faculty of Health and Sport Science (FCSD), Huesca, Spain; 10 CIBER of Obesity and Nutrition (CIBERobn), Madrid, Spain; 11 Instituto Internacional de Investigación e Innovación en Envejecimiento, Universidad de Extremadura, Cáceres, Spain; 12 Foundation for Biomedical Research, Getafe University Hospital. Getafe, Spain; 13 ImFINE Research Group, Universidad Politécnica de Madrid. Madrid, Spain

**Keywords:** Chronic diseases, Ageing, Sedentary time, Anxiety, Quality of life

## Abstract

**OBJECTIVES:**

This study investigated the associations of chronic diseases with changes in lifestyle and health behaviours in older people following the coronavirus disease 2019 (COVID-19) lockdown in Spain and compared the differences in changes over time.

**METHODS:**

1,092 participants (80.3±5.6 years; 66.5% female) from 2 Spanish cohorts were included. Telephone-based questionnaires were conducted to evaluate lifestyle and health risk behaviours at the end of lockdown and 7 months post-lockdown. Participants were classified as having physician-diagnosed chronic diseases based on self-reported data. Cox proportional models adjusted for major confounders were used.

**RESULTS:**

Compared to those without the corresponding chronic diseases, older people with hypertension were less likely to report increased alcohol consumption (hazard ratio [HR], 0.73; 95% confidence interval [CI], 0.55 to 0.99). Pulmonary diseases were associated with lower risks of increased sedentary time (HR, 0.58; 95% CI, 0.39 to 0.86) and worsened sleep quality (HR, 0.56; 95% CI, 0.36 to 0.87), while cardiovascular diseases were associated with a lower risk of decreased sedentary time (HR, 0.58; 95% CI, 0.38 to 0.88). Depression was linked to a higher likelihood of improved diet quality (HR, 1.53; 95% CI, 1.00 to 2.36). Cancer pacients were less likely to have worsened sleep quality (HR, 0.44; 95% CI, 0.22 to 0.89) but more likely to have reduced their frequency of social contact (HR, 2.05; 95% CI, 1.05 to 3.99).

**CONCLUSIONS:**

Older people with chronic diseases showed beneficial changes in lifestyle and health risk behaviours after the COVID-19 lockdown. In particular, older people with hypertension, pulmonary disease, and cancer tended to make beneficial lifestyle and health behaviour changes. However, older people with cardiovascular disease and depression engaged in more health risk behaviours.

## GRAPHICAL ABSTRACT

[Fig f2-epih-44-e2022026]

## INTRODUCTION

The coronavirus disease 2019 (COVID-19) pandemic has forced many national governments to implement social distancing measures. On March 15, 2020, the government of Spain approved a strict lockdown period to fight the spread of the virus, during which the population was instructed to stay at home, with only outings for basic necessities such as shopping or going to the hospital being permitted [1]. Starting on May 2, 2020, some restrictions were gradually lifted to return to the “new normal,” beginning with permission to leave the house to exercise or walk, and on June 23, 2020, the strong recommendation to avoid personal contact was lifted [2]. Nevertheless, since the lockdown period required radical changes in lifestyle among the population that interrupted normal daily activities [3], adverse health effects could also be expected. A lockdown could therefore result in an increased prevalence of health risk behaviours [4-6] and potentially have a long-term negative health impact on people with non-communicable diseases [7]. Therefore, this situation is particularly relevant and concerning for people with comorbidities, for whom physical activity, nutrition, cognitive training, and management of metabolic and vascular risk factors are essential for controlling symptoms and reducing the incidence of chronic diseases [8,9]. Since most older people have comorbidities, they are considered vulnerable to lockdown measures [10,11]. Nonetheless, to our knowledge, no studies have yet examined the effects of this lockdown on health risk behaviours during the return to the “new normal” among older people with various chronic diseases, even though lockdowns could jeopardise the sustainability of healthcare systems by worsening the condition of this specific population [7].

Thus, the main aim of this study was to assess the associations of hypertension, musculoskeletal disease, pulmonary disease, cardiovascular disease (CVD), depression, and cancer with the lifestyle and health behaviours of older people in Spain following the strict 2-month lockdown compared to older people without those chronic diseases. In addition, the secondary aim was to evaluate the differences in these changes from the time of the lockdown until the return to the “new normal.”

## MATERIALS AND METHODS

### Study design and cohorts

A new COVID-19 sub-cohort based on 2 different Spanish prospective cohorts was created for this study. First, the Toledo Study for Healthy Ageing (TSHA) was a prospective study of community-dwelling individuals aged ≥ 65 years from the province of Toledo that was conducted across 3 waves between 2006 and 2009, 2011 and 2013, and 2016 and 2017. Second, the elderly EXERNET multi-center study included non-institutionalised individuals aged ≥ 65 years recruited in Aragón, Castilla-La Mancha, Cádiz, and Madrid. This study was also conducted across 3 waves around the same time, from 2008 to 2009, 2011 to 2012, and 2016 to 2017.

For this prospective study, baseline data were collected between April 28 and June 30, 2020, while follow-up was conducted in December 2020. First, a total of 2,982 participants were recruited from both cohorts (TSHA and EXERNET). Of these participants, 589 individuals could not be contacted, 605 declined to participate, and 1,788 agreed to participate (938 from TSHA and 850 from EXERNET; 63% total response rate). After follow-up, 217 individuals could not be contacted, 324 refused to participate, and 1,247 agreed to participate (688 from TSHA and 559 from EXERNET; 70% total response rate). Finally, 1,092 participants completed the second assessment (66.5% of whom were females) and were included in the analyses after excluding those who had been infected with COVID-19. Thus, this sub-cohort included subjects who had been examined during the COVID-19 lockdown in Spain and 8-month after the lockdown.

### Outcomes and exposure variables

The participants completed a telephone-based structured interview to collect data on health behaviours, mental and physical health, and their potential determinants, including demographic and social variables at baseline and follow-up. The outcomes were health risk factors and lifestyle changes that may have been affected by lockdown. The particular outcomes in the present study were changes in alcohol consumption, diet quality (based on the 14-point Mediterranean Diet Adherence Screener Questionnaire) [12], weight, total minutes of daily sedentary time (watching TV, using electronic devices, reading, listening to music, napping, and sunbathing), physical activity (based on the Physical Activity Scale for the Elderly) [13], hours of night-time sleep, sleep quality (with possible responses of “excellent,” “good,” “fair,” “poor,” and “very poor”), anxiety (based on the 12-item General Health Questionnaire [GHQ-12]) [14], frequency of social contact (daily socialisation with family or friends), whether the participant lived alone, and quality of life (based on the 12-item Short-Form Health Survey, distinguishing between the physical component summary [PCS] and the mental component summary [MCS]) [15]. Longitudinal changes in these variables were assessed to categorise the participants according to their post-lockdown changes using the cut-off points indicated in Supplementary Material 1.

The exposure variables included self-reported, physician-diagnosed chronic conditions: hypertension (n=727), musculoskeletal disease (n=665), pulmonary disease (n=217), CVD (n=243), depression (n=172), and cancer (n=111). Some participants had multiple conditions.

### Other variables

The following variables were also recorded and treated as potential confounders: sex, age, education level (illiterate, primary school, secondary school, university), individual income (≤ 600, > 600 to < 900, and ≥ 900 EUR/mo), marital status (single, married/living together, divorced/separated, widowed), and the presence of any other chronic diseases aside from those included as exposure variables (hypertension, musculoskeletal disease, pulmonary disease, CVD, depression, and cancer).

### Statistical analysis

The normal distribution of the variables was determined using the Kolmogorov-Smirnov test and normal probability plots. The characteristics of the study groups and the differences between them at baseline and follow-up were determined through basic descriptive tests (means and standard deviations or the prevalence [%] of participants in that category) and the paired 2-sample t-test. The relationships between chronic diseases and health risk behaviours and lifestyle changes were investigated using Cox proportional hazard models, with follow-up time as a time-varying covariate. Major confounders were also used as covariates. The results were reported as hazard ratios (HRs) and their 95% confidence intervals (CIs). In all analyses, subjects whose behaviours remained unchanged between the lockdown and post-lockdown periods were used as reference groups. In addition, posterior sensitivity analyses were conducted for anxiety, particularly for those with depression and cancer since the results related to these participants were inconclusive. To better understand changes in anxiety in relation to each of the chronic diseases included in the analysis, multivariable logistic regression was conducted to estimate odds ratios (ORs) and their respective 95% CIs, again using major confounders as covariates. The differences between participants with and without any particular chronic disease who completed the second assessment and who were lost to follow-up were also determined through basic descriptive tests (means and standard deviations or prevalence [%] of participants in a given group) and the independent samples t-test (Supplementary Materials 2 and 3). Statistical analyses were performed using the SPSS version 24 (IBM Corp., Armonk, NY, USA). Statistical significance was set at p-value ≤ 0.05.

### Ethics statement

The Clinical Research Ethics Committee of the Toledo Hospital Complex (protocol #2203/30/2005) and the Clinical Research Ethics Committee of Aragón (#18/2008) approved the TSHA and EXERNET, respectively. In addition, all participants gave verbal informed consent.

## RESULTS

Table 1 summarizes the main characteristics of the participants according to the presence of chronic diseases and differences between lockdown and the “new normal.” In summary, the diet quality of those with hypertension, CVD, musculoskeletal disease, and depression significantly improved at follow-up. Physical activity significantly increased among all participants with chronic diseases. In addition, those with hypertension and CVD significantly decreased their amounts of sedentary time. Moreover, significant weight loss was observed among those with hypertension and cancer. Finally, a significant decline in PCS scores was observed in all participants with chronic diseases except for those with cancer.

Supplementary Material 2 shows the main characteristics of and differences between participants with and without chronic disease during the lockdown. Participants with hypertension had significantly higher weights, PCS scores, and MCS scores than those without hypertension. Those with pulmonary diseases also had worse GHQ-12 scores than those without pulmonary diseases. Participants without depression, CVD, and musculoskeletal diseases had significantly better PCS, MCS, and GHQ-12 scores than participants with those conditions. Participants without cancer tended to be significantly older and have significantly better PCS scores compared to those with cancer. Supplementary Material 3 summarizes the main characteristics of and differences between the participants who completed the second assessment and those who were lost to follow-up, showing that participants who did not complete both assessments were significantly older, less sedentary, and had worse PCS and MCS scores. The primary reasons for not participating in the second interview were outright refusal without explanation (40%), a lack of time (18%), and health-related conditions (11%).

**Figure 1.**
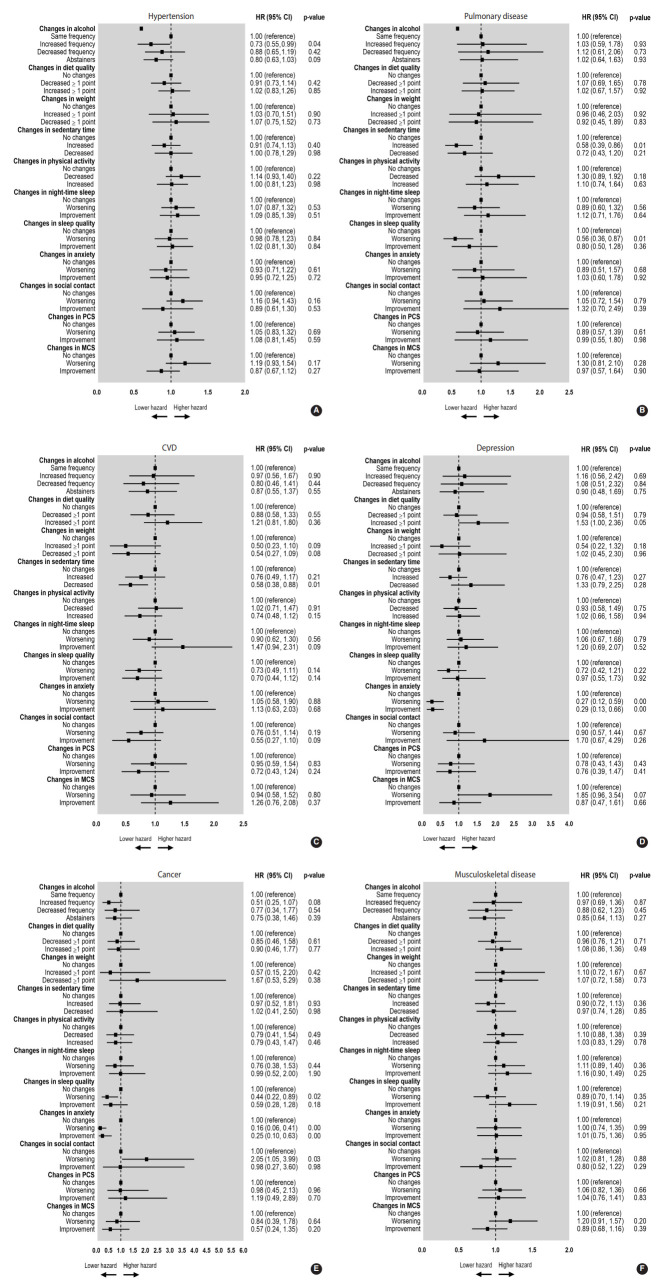
Forest plot showing the relationships between different chronic diseases (A: hypertension, B: pulmonary diseases, C: cardiovascular diseases (CVD), D: depression, and E: cancer, and F: musculoskeletal diseases) and changes in health risk behaviours and lifestyle after the coronavirus disease 2019 lockdown period. Squares and bars represent HRs and corresponding 95% CIs of changes. Data in cursive show tendencies. ST, sedentary time; PCS, physical component summary of the 12-Item Short-Form Health Survey; MCS, mental component summary of the 12-Item Short-Form Health Survey; HR, hazard ratio; CI, confidence interval. HRs were adjusted for baseline age, sex (male or female), education level (illiterate, primary, secondary, or university), marital status (single, married, divorced, widowed), and income (≤600, >600 to ≤900, >900 EUR/mo).

The relationship between chronic diseases and changes in health risk behaviours and lifestyle among those with and without chronic diseases are shown in Figure 1. Increased frequency of alcohol consumption was 0.73 times lower among participants with hypertension than among participants without hypertension (Figure 1A). Increased sedentary time and worsened sleep quality were 0.58 times and 0.56 times less likely among participants with pulmonary diseases than among those without pulmonary diseases (Figure 1B). A decrease in sedentary time among those with CVD was 0.58 times less frequent than among participants without CVD (Figure 1C). Among participants with depression (Figure 1D), a 1.53-fold higher likelihood of improved diet quality was observed compared to those without depression, and their anxiety level was more likely to be stable—with HRs of 0.27 and 0.29 for worsening and improvement, respectively—than those of older people without depression. Moreover, participants with depression were 1.85 times less likely to have a worsened average MCS score than those without depression. Similarly, worsening sleep quality, reduced frequency of social contact, worsening of anxiety, and improvement of anxiety were 0.44, 2.05, 0.16, and 0.25 times lower, respectively, for those with cancer than those without cancer (Figure 1E). No significant association was found concerning musculoskeletal diseases (Figure 1F). When sensitivity analyses were undertaken to examine changes in anxiety among participants with depression and cancer, worsened anxiety was more likely among those with depression (OR, 2.19; 95% CI, 1.02 to 4.69), although no significant results were found for older people with cancer.

## DISCUSSION

This study examined the associations of chronic diseases with lifestyle and health behaviour changes during the return to the “new normal” after strict lockdown measures in response to the COVID-19 pandemic, as well as differences in lifestyle and health behaviours between during and after the lockdown. In general, we observed improvements related to diet quality, physical activity, sedentary time, and weight among all participants with chronic diseases, although all of the participants with chronic diseases (except for cancer) also had worse PCS scores than those with no chronic diseases. The differences between those who completed the survey at both baseline and follow-up and those lost to follow-up may be explained by a phenomenon observed in a study by Wagner et al. [16], in which poor physical (e.g., hearing or visual impairments) and mental (e.g., impaired memory or cognitive ability) health were found to prevent some older people from participating in surveys. Nonetheless, although the participants’ lifestyles improved during the return to the “new normal,” lockdown had different medium-term health effects depending on the participants’ particular chronic diseases compared to their counterparts without chronic conditions. Our findings showed that hypertension, pulmonary disease, and cancer were associated with positive changes in health behaviour compared to those without these conditions, while CVD and depression were associated with lifestyle and behavioural changes that increased participants’ health risks. However, musculoskeletal diseases did not appear to have any influence, when comparing those with and without musculoskeletal conditions. Overall, health risk behaviours decreased and lifestyles improved after Spain’s strict, 2-month lockdown, resulting in drastic lifestyle changes among older people with chronic diseases.

Hypertension was associated with a lower risk of increased alcohol consumption, indicating a decrease in adverse health behaviours among those with hypertension. A decrease in the frequency of alcohol consumption could lead to a considerable reduction in the risk of all-cause and cardiovascular death among older people with hypertension [17,18]. This is especially important in the context of the COVID-19 pandemic since Browne et al. [19] found that older people with hypertension were more vulnerable to cardiometabolic disturbance cascades during the COVID-19 pandemic, which could increase their risk of CVD and metabolic diseases. Therefore, the participants with hypertension appear to have shown substantial concern for their health in response to the COVID-19 pandemic through the improvement of this particular risk factor following the lockdown compared to their counterparts without hypertension. In addition, a significant improvement in diet quality was also observed among older people with hypertension, which is closely related to alcohol consumption as well as the other 2 main health-related lifestyle components (physical activity and sedentary time). Likewise, older people with pulmonary diseases also showed a reduction in adverse health behaviours, as indicated by their lower risk of increased sedentary time and poor sleep quality than those without pulmonary diseases. These health benefits are essential among this population since COVID-19 causes respiratory problems, and those with pulmonary diseases are at a particularly increased risk of COVID-19 morbidity compared to those with metabolic conditions alone [20]. Therefore, it makes sense that older peoplewith pulmonary diseases comprised one of the most health-conscious populations in terms of lifestyle and health behaviour improvements following Spain’s lockdown. Among participants with cancer, more health benefits than risks were also observed, including a lower risk of poor sleep quality and a higher likelihood of frequent social contact. Although feelings of loneliness, a lack of social support, and isolation due to COVID-19 among individuals with chronic diseases have been associated with reductions in physical activity [21,22], older people with cancer from our sample did not show significant changes in physical activity compared to those without cancer. Furthermore, the incidence rate of sleep disorders among cancer patients ranged from 30% to 93%, which is considerably higher than that of the general population (9-33%) [23]. Since sleep disorders are associated with detrimental effects on health outcomes and can impact psychological factors, functional status, and the use of drugs and can decrease quality of life [24], the decrease in the likelihood of poor sleep quality observed in this study is highly positive. Finally, our results related to anxiety were inconclusive, showing both a lower risk of increased and decreased anxiety among older people with cancer compared to their counterparts without cancer. The specific characteristics and factors affecting each subject should be examined in future studies to better understand the degree to which anxiety and mental health are affected among this population [25]. Even so, although it was not statistically significant, the MCS scores of older people with cancer had improved at the time of the follow-up.

Conversely, CVD and depression were associated with a decline in health risk behaviours during the return to the “new normal.” Although older people with CVD engaged in less sedentary behaviours by the time of the follow-up, they showed a lower likelihood of decreased sedentary time compared to those without CVD. This indicates that older people with CVD may not have made positive health behaviours or lifestyle changes related to sedentary time. This failure to reduce sedentary time after lockdown could be particularly detrimental to individuals with CVD since sedentary time is associated with increased CVD complications [26] and a high risk of all-cause and CVD mortality [27]. Similarly, regular exercise is associated with many health-related factors, including a reduced risk of future cardiac dysfunction [28]. Older people with depression were more likely to have a low MCS score compared to those without depression, although they also had a higher likelihood of increasing the quality of their diet, which is widely known to be a potential health-protective factor [29]. Nevertheless, given the characteristics of depression, poor mental health indicated by low MCS scores following lockdown conditions could exacerbate the condition of individuals with depression to a great extent. Recent studies have examined the relevance of pre-existing mental health comorbidities when coping with the effects of the COVID-19 pandemic [30], and their findings have indicated that the COVID-19 pandemic constituted a stressful and uncontrollable life event that may have worsened the mental health of many older adults [31]. Additionally, participants with depression were also found to be at risk of increased anxiety, which also negatively affects depression. Some of the reasons for the decline in mental health may be due to delays in the delivery of psychotropic medications, a lack of access to primary care or outpatient clinics, increased financial difficulty, personal concern about contracting COVID-19, spending a long period of time at home, and decreased living conditions [32].

Finally, musculoskeletal diseases were not associated with any changes in health risks after lockdown, likely because the change in the level of physical activity and strength training, which are determinants of this disease [33], during the return to the “new normal” was roughly the same for those with and without musculoskeletal disorders. Therefore, understanding the determinants of health risk behaviours and lifestyle during the COVID-19 pandemic is crucial when developing public health interventions [34], especially those targeted to individuals with CVD and cancer, as they were negatively affected the most by the COVID-19 pandemic in the medium term. In addition, it is important to understand that, although the lifestyles of individuals with chronic diseases may have improved during the return to the “new normal,” these improvements may not have been as consequential compared to the changes made by individuals without chronic diseases. Lifestyle improvements as a result of the COVID-19 lockdown could help improve the sustainability of healthcare systems, which is a vital element for the care of those with chronic diseases, particularly patients with comorbidities [7,35,36]. Solutions must be developed to prevent potentially devastating, long-term negative health effects for those with multiple or more severe diseases that require routine symptom monitoring and complex drug regimens [7].

Similar to the results of previous studies, we also found that individuals with chronic diseases had significantly worse PCS and MCS scores compared to their counterparts without chronic diseases.

This study is not without limitations. The results may not be generalisable to the worldwide population due to the particularly strict lockdown measures implemented in Spain during the COVID-19 pandemic. Furthermore, the relatively small sample size of individuals with cancer means that the results concerning this group should be interpreted with caution, and future studies should examine the effects of a lockdown on lifestyle and health risk behaviours among a larger group of cancer patients to better understand the results. In addition, the variables were collected using subjective data from structured telephone interviews, and chronic diseases were self-reported; thus, the prevalence of some of the diseases may have been underestimated. However, most of the questions were taken from validated questionnaires [12-15,37], and older peopleare very heavy users of health services. Therefore, under-diagnosis was unlikely for this reason. Similarly, before the COVID-19 pandemic, the participants had already completed an interview at home as members of their respective cohorts. Therefore, they were already aware of the questions and the process, thus reducing the risk of reporting bias. To our knowledge, this was the first study to investigate how strict lockdown measures affected lifestyle changes during Spain’s return to the “new normal” among a relatively large sample of older people with various chronic diseases. In addition, participants who were institutionalised or infected with COVID-19 were excluded to ensure the homogeneity of the sample.

In conclusion, our results show evidence of beneficial changes in health risk behaviours and lifestyle after the COVID-19 lockdown among older people with chronic diseases. However, when compared to older people without chronic diseases, those with hypertension, pulmonary diseases, and cancer showed the highest benefits. Older people with CVD and depression seem to have experienced some worsened health risk behaviours and lifestyle changes that could have affected them more negatively. Musculoskeletal diseases did not appear to have any effect when comparing those with and without musculoskeletal conditions. Therefore, these findings suggest that public health interventions should be developed to manage the COVID-19 pandemic and future similar situations to prevent dangerous long-term effects on the health of older people, with a particular focus on CVD and depression.

## Figures and Tables

**Figure f2-epih-44-e2022026:**
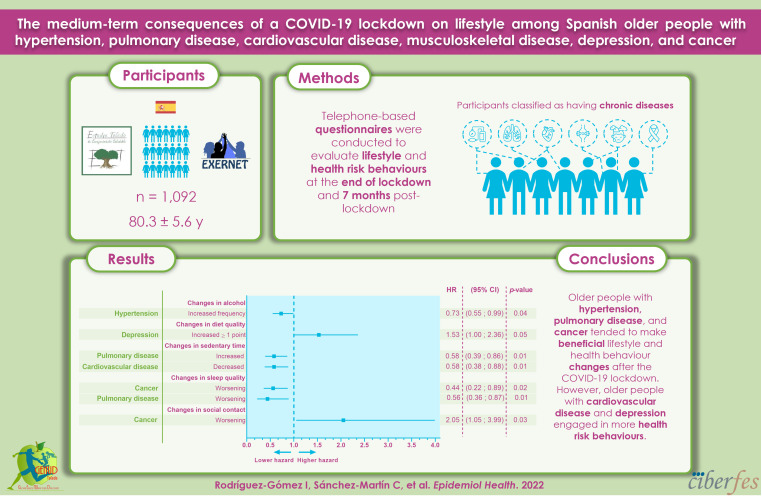


**Table 1.** Socio-demographic, lifestyle, and health-related characteristics of the study population during and after the coronavirus disease 2019 lockdown stratified by chronic diseases (hypertension, depression, cancer, pulmonary disease, CVD, and musculoskeletal disease)^1^

Characteristics			Total participants		Hypertension		Pulmonary disease		CVD		Musculoskeletal disease		Depression		Cancer	
			During (n=1,092)	After (n=1,092)	During (n=727)	After (n=727)	During (n=217)	After (n=217)	During (n=243)	After (n=243)	During (n=665)	After (n=665)	During (n=172)	After (n=172)	During (n=111)	After (n=111)
Socio-demographic variables																
	Age (yr)		80.3±5.6		80.6±5.6		81.0±5.8		81.5±5.6		81.0±5.6		79.9±5.6		79.6±4.8	
	Female		66.5		68.1		70.5		63.0		78.6		83.7		57.7	
	Education															
		Illiterate	14.7		17.7		12.9		12.3		17.3		22.7		11.7	
		Primary	55.9		56.4		59.0		58.0		58.5		57.6		48.6	
		Secondary	13.3		11.3		12.9		13.6		10.1		9.3		14.4	
		University	8.2		7.8		9.7		9.5		5.1		3.5		12.6	
	Marital status															
		Single	4.0		3.6		1.4		3.7		2.7		2.9		1.8	
		Married	57.9		55.8		54.4		54.7		52.3		51.7		58.6	
		Divorced	2.3		1.9		3.2		2.5		2.1		1.2		1.8	
		Widowed	35.7		38.7		41.0		39.1		42.7		44.2		37.8	
	Income (EUR/mo)															
		≤600	22.6		22.6		24.9		22.2		26.8		26.7		15.3	
		>600-≤900	29.2		30.9		30.4		28.4		30.2		34.3		27.0	
		>900	31.7		30.3		30.9		32.1		23.6		24.4		35.1	
	Lived alone		27.7	29.1	28.7	29.8	25.8	27.2	26.3	28.8	31.6	32.8	31.4	29.1	24.3	27.9
	Daily socialization		90.8	74.6	91.2	74.1	89.9	73.3	91.8	77.8	92.3	75.9	93.6	77.3	91.9	79.3
Lifestyle-behaviours																
	Smokers		2.7	3.2	2.2	2.5	2.3	4.1	2.5	3.3	1.2	1.8	1.7	1.7	1.8	1.8
	Alcohol intake (days/wk)															
		Daily	19.5	21.9	17.9	20.1	17.1	19.4	17.3	19.3	15.2	17.0	11	16.3	23.4	27.9
		3-5	3.9	3.8	3.7	3.3	4.1	3.2	2.9	3.3	3.0	2.9	2.9	2.9	3.6	4.5
		1-2	3.2	5.2	2.5	4.3	4.1	4.1	3.3	2.5	3.2	3.9	4.1	3.5	7.2	3.6
		<1	8.8	5.7	9.8	5.2	7.4	4.1	8.6	5.8	10.2	5.6	12.2	4.7	9.0	6.3
		Non-drinker	59.0	61.7	60.7	65.2	59.9	66.8	62.1	67.9	62.0	68.6	61	71.5	51.4	56.8
		Stopped recently	5.6	1.6	5.5	1.9	7.4	2.3	5.8	1.2	6.5	2.0	8.7	1.2	5.4	0.9
	MEDAS index		7.0±1.8	7.2±1.7^*^	7.0±1.7	7.2±1.7^*^	6.8±1.7	7.0±1.7	6.9±1.7	7.2±1.7^*^	6.9±1.7	7.1±1.7^*^	6.8±1.8	7.3±1.7^*^	7.1±1.8	7.2±1.8
	PASE score		72.2±45.2	82.8±52.6^*^	69.5±43.9	79.3±50.7^*^	66.5±44.9	78.1±52.5^*^	63.9±44.9	72.5±50.4^*^	67.9±43.6	77.6±51.8^*^	(65.2±39.7)	76.4±53.7^*^	66.4±46.4	79.0±46.7^*^
	Weight (kg)		70.6±12.1	70.5±12.2^*^	71.8±12.6	71.4±12.3^*^	71.7±10.5	70.5±11.6	69.4±12.4	70.3±13.2	69.7±12.0	69.7±12.2^*^	68.1±11.0	68.7±10.6	73.4±10.7	72.6± 11.1^*^
	Height (m)		1.6±0.2		1.6±0.2		1.6±0.2		1.6±0.2		1.5±0.2		1.5±0.1		1.6±0.9	
	Total ST (min/day)		423.3±182.7	399.9±202.1^*^	425.6±184.6	400.0±202.1^*^	407.1±176.9	405.7±209.8	453.1±186.7	413.0±198.0^*^	413.0±179.0	399.6±207.7	409.5±181.0	378.5±178.6	445.4±174.3	424.5±206.2
Sleep characteristics																
	Hours of night-time sleep															
		Short sleep (≤6 hr)	31.5	33.7	32.2	34.0	34.1	36.4	32.9	35.0	33.7	34.4	23.8	26.7	38.7	30.6
		Normal sleep	50.6	42.5	50.1	40.6	48.8	38.2	48.6	40.3	48.3	40.3	52.3	41.9	42.3	42.3
		Long sleep (≥9 hr)	17.1	18.0	16.9	18.6	15.7	18.4	17.7	19.3	17.1	18.3	23.3	25.0	18.0	22.5
	Overall sleep quality															
		Very good	6.3	5.5	4.8	4.1	4.1	5.1	4.1	5.8	3.6	3.8	2.9	5.8	6.3	4.5
		Good	54.0	50.1	52.4	48.6	47.9	41.9	49.8	41.6	50.4	43.2	46.5	36.6	48.6	41.4
		Fair	20.1	21.6	21.5	22.4	21.2	24.0	20.6	20.6	22.3	25.1	23.8	26.7	20.7	19.8
		Poor	4.0	4.9	4.3	5.5	5.1	5.1	4.5	5.8	5.1	6.5	4.7	6.4	4.5	9.0
		Very poor	1.3	0.7	1.7	0.7	1.8	0.5	2.1	1.2	1.8	1.1	1.7	1.2	1.8	0.9
Health-related variables																
	SF-12, PCS		47.1±10.4	44.0±12.2^*^	46.2±10.9	42.5±12.7^*^	45.7±11.3	41.9±13.0^*^	43.8±12.9	40.4±13.1^*^	44.5 ±11.1	40.7±13.0^*^	43.2±11.5	41.3±13.9^*^	44.2±13.0	41.6±14.3
	SF-12, MCS		53.5±9.3	52.9±9.9	53.4±9.8	52.7±10.5	53.6±10.3	50.6±11.3	52.9±10.6	52.3±11.2	53.4 ±9.7	52.7±10.4	51.1±11.3	48.4±12.9	54.5±10.1	53.4±10.4
	GHQ score		9.2±3.8	9.3±4.0	9.4±3.9	9.6±4.2	9.8±4.7	10.2±4.5	10.0±4.6	10.0±4.7	9.7±4.1	9.8±4.3	11.1±5.2	11.6±5.4	9.8±4.4	9.8±4.2
Days elapsed^2^			214.0±9.1		214.3±8.9		213.6±9.0		213.2±9.1		214.3±9.2		214.1±8.9		213.4±8.6	

Variables are presented as mean±standard deviations or % of participants in that category. CVD, cardiovascular disease; SD, standard deviation; MEDAS, Mediterranean Diet Assessment Score; PASE, Physical Activity Scale for the Elderly; ST, sedentary time; SF-12, 12-Item Short-Form Health Survey; PCS, physical component summary of the SF-12; MCS, mental component summary of the SF-12; GHQ, General Health Questionnaire.

1 Higher scores for the MCS and PCS of the SF-12, the PASE, and the MEDAS, and lower scores on the GHD indicate better health.

2 Data was collected at the end of the lockdown period and 7 months later.

* p<0.05 in the paired sample t-test for change values post-lockdown.

## SUPPLEMENTARY MATERIALS

Supplementary Material 1. Classification of change in lifestyle, and health-related characteristics.

Supplementary Material 2. Socio-demographic, lifestyle, and health-related characteristics of the study population during COVID 19 lockdown stratified by the presence of chronic diseases (hypertension, depression, cancer, and pulmonary, cardiovascular, and musculoskeletal diseases).

Supplementary Material 3. Socio-demographic, lifestyle, and health-related characteristics of the study population during COVID 19 lockdown comparing the participants who had completed the 2nd assessment and those lost to follow-up.
