# Supplementary material for: The medium-term consequences of a COVID-19 lockdown on lifestyle among Spanish older people with hypertension, pulmonary disease, cardiovascular disease, musculoskeletal disease, depression, and cancer
Source: Epidemiol Health. 2022 Feb 21;44:e2022026. doi: 10.4178/epih.e2022026 (PMC9684008; doi:10.4178/epih.e2022026)
Supplement: Supplementary Material 1. — Classification of change in lifestyle, and health-related characteristics. [file epih-44-e2022026-suppl1.docx]

| **Supplementary Material 1.** Classification of change in lifestyle, and health-related characteristics. | | | | | |
| --- | --- | --- | --- | --- | --- |
|  | **Scale** | **Range** | **Interpretation** | **Classification of change^§^** | |
| Alcohol consumption |  |  |  | Same | Drinkers who did not change their frequency of consumption |
|  |  |  |  | Increased | Drinkers who increased their frequency of consumption |
|  |  |  |  | Decreased | Drinkers who decreased their frequency of intake |
|  |  |  |  | Abstainers | Not-drinkers who maintained their non-drinking status after confinement |
|  |  |  |  |  |  |
| Diet quality | MEDAS | 0-12 | ↑ score  ↑ adherence to the Mediterranean diet | No changes | Change = 0 |
|  |  |  |  | Worsening | Change ≤ -1 point |
|  |  |  |  | Improvement | Change ≥ 1 point |
|  |  |  |  |  |  |
| Weight |  |  |  | Maintained | Change ≤ 1kg |
|  |  |  |  | Increased | Change > 1kg |
|  |  |  |  | Decreased | Change < 1kg |
|  |  |  |  |  |  |
| Sedentary time |  |  |  | Average | Change between the 75^th^ and the 25^th^ percentile |
|  |  |  |  | Unhealthier | Increased sedentary time more than the 75^th^ percentile |
|  |  |  |  | Healthier | Increased sedentary time more than the 25^th^ percentile |
|  |  |  |  |  |  |
| Physical activity | PASE | >0 | ↑ score  ↑ physical activity | Average | Change between the 75^th^ and the 25^th^ percentile |
|  |  |  |  | Unhealthier | Increased physical activity more than the 75^th^ percentile |
|  |  |  |  | Healthier | Increased physical activity more than the 25^th^ percentile |
|  |  |  |  |  |  |
| Night-time sleep |  |  |  | Average | Stayed in the same normal/non-normal sleep category |
|  |  |  |  | Worsening | Change from normal to short or long sleep |
|  |  |  |  | Improvement | Change form short or long sleep to normal sleep |
|  |  |  |  |  |  |
| Sleep quality | “Overall sleep quality” score | 1-5 | ↑ score  ↓ sleep quality | Average | No changes in the “poor sleep quality” score |
|  |  |  |  | Worsening | Increase in the “poor sleep quality” score |
|  |  |  |  | Improvement | Decrease in the “poor sleep quality” score |
|  |  |  |  |  |  |
| Anxiety | GHQ | 0-36 | ↑ score  ↑ anxiety conditions | No changes | Change = 0 |
|  |  |  |  | Worsening | Change ≥ 1point |
|  |  |  |  | Improvement | Change ≤ -1point |
|  |  |  |  |  |  |
| Social contact |  |  |  | No changes | Maintained the same social contact |
|  |  |  |  | Worsening | Decreased their social contact |
|  |  |  |  | Improvement | Increased their social contact |
|  |  |  |  |  |  |
| PCS | SF-12 | 0-100 | ↑ score  ↑ quality of life | No changes | -4.99 ≤ change ≥ 4.99 points |
|  |  |  |  | Worsening | Change ≥ 5 points |
|  |  |  |  | Improvement | Change ≤ -5 points |
|  |  |  |  |  |  |
| MCS | SF-12 | 0-100 | ↑ score  ↑ quality of life | No changes | -4.99 ≤ change ≥ 4.99 points |
|  |  |  |  | Worsening | Change ≥ 5 points |
|  |  |  |  | Improvement | Change ≤ -5 points |
|  |  |  |  |  |  |
| Abbreviations: MEDAS, Mediterranean Diet Assessment Score; PASE, Physical Activity Scale for the Elderly; GHQ, General Health Questionnaire; SF-12, 12-Item Short-Form Health Survey; PCS, Physical Component Score of the SF-12; MCS, Mental Component Score of the SF-12. § Change for all variables was calculated as the score post-quarantine minus the score during lockdown. | | | | | |
