# Supplementary material for: The medium-term consequences of a COVID-19 lockdown on lifestyle among Spanish older people with hypertension, pulmonary disease, cardiovascular disease, musculoskeletal disease, depression, and cancer
Source: Epidemiol Health. 2022 Feb 21;44:e2022026. doi: 10.4178/epih.e2022026 (PMC9684008; doi:10.4178/epih.e2022026)
Supplement: Supplementary Material 2. — Socio-demographic, lifestyle, and health-related characteristics of the study population during COVID 19 lockdown stratified by the presence of chronic diseases (hypertension, depression, cancer, and pulmonary, cardiovascular, and musculoskeletal diseases). [file epih-44-e2022026-suppl2.docx]

| **Supplementary Material 2.** Socio-demographic, lifestyle, and health-related characteristics of the study population during COVID 19 lockdown stratified by the presence of chronic diseases (hypertension, depression, cancer, and pulmonary, cardiovascular, and musculoskeletal diseases). | | | | | | | | | | | | | | |
| --- | --- | --- | --- | --- | --- | --- | --- | --- | --- | --- | --- | --- | --- | --- |
|  | **Hypertension** | | | | **Pulmonary disease** | | | | **CVD** | | | | | |
|  | **With**  (n=727) | | **Without**  (n=360) | | **With**  (n=217) | | **Without**  (n=875) | | **With**  (n=243) | | | **Without**  (n=849) | | |
| **Socio-demographic variables** |  | |  |  |  |  |  |  |  | | | | | |
| **Age, years**; mean (SD) | 80.6 | (5.6) | 79.7 | (5.5) | 81.0 | (5.8) | 80.2 | (5.5) | 81.5 | | (5.6) | 80.1 | | (5.5) |
| **Female**; % | 68.1 | | 63.6 | | 70.5 | | 65.5 | | 63.0 | | | 67.5 | | |
| **Education**; % |  | |  | |  | |  | |  | | |  | | |
| Illiterate | 17.7 | | 10.0 | | 12.9 | | 15.2 | | 12.3 | | | 15.4 | | |
| Primary | 56.4 | | 55.3 | | 59.0 | | 55.1 | | 58.0 | | | 55.2 | | |
| Secondary | 11.3 | | 16.7 | | 12.9 | | 13.4 | | 13.6 | | | 13.2 | | |
| University | 7.8 | | 9.2 | | 9.7 | | 7.9 | | 9.5 | | | 7.9 | | |
| **Marital status**; % |  | |  | |  | |  | |  | | |  | | |
| Single | 3.6 | | 5.0 | | 1.4 | | 4.7 | | 3.7 | | | 4.1 | | |
| Married | 55.8 | | 62.2 | | 54.4 | | 58.8 | | 54.7 | | | 58.8 | | |
| Divorced | 1.9 | | 3.1 | | 3.2 | | 2.1 | | 2.5 | | | 2.2 | | |
| Widowed | 38.7 | | 29.4 | | 41.0 | | 34.4 | | 39.1 | | | 34.8 | | |
| **Income**; % |  | |  | |  | |  | |  | | |  | | |
| ≤600€ per month | 22.6 | | 22.5 | | 24.9 | | 22.1 | | 22.2 | | | 22.7 | | |
| >600≤900€ per month | 30.9 | | 25.6 | | 30.4 | | 28.9 | | 28.4 | | | 29.4 | | |
| >900€ per month | 30.3 | | 34.7 | | 30.9 | | 31.9 | | 32.1 | | | 31.6 | | |
| **Living alone; %** | 28.7 | | 25.6 | | 25.8 | | 28.1 | | 26.3 | | | 28.0 | | |
| **Daily socialization; %** | 91.2 | | 90.3 | | 89.9 | | 91.1 | | 91.8 | | | 90.6 | | |
| **Lifestyle-behaviours** |  | | | |  | | | |  | | | | | |
| **Smokers; %** | 2.2 | | 3.6 | | 2.3 | | 2.7 | | 2.5 | | | 2.7 | | |
| **Alcohol intake; %** |  | | | |  | | | |  | | | | | |
| Daily | 17.9 | | 23.1 | | 17.1 | | 20.1 | | 17.3 | | | 20.1 | | |
| 3-5 days per week | 3.7 | | 4.4 | | 4.1 | | 3.9 | | 2.9 | | | 4.2 | | |
| 1-2 days per week | 2.5 | | 4.7 | | 4.1 | | 3.0 | | 3.3 | | | 3.2 | | |
| Less than 1 day per week | 9.8 | | 6.9 | | 7.4 | | 9.1 | | 8.6 | | | 8.8 | | |
| Non-drinker | 60.7 | | 55.0 | | 59.9 | | 58.7 | | 62.1 | | | 58.1 | | |
| Stopped recently | 5.5 | | 5.8 | | 7.4 | | 5.1 | | 5.8 | | | 5.5 | | |
| **MEDAS index; mean (SD)** | 7.0 | (1.7) | 7.1 | (1.8) | 6.8 | (1.7) | 7.1 | (1.8) | 6.9 | | (1.7) | 7.0 | (1.8) | |
| **PASE score; mean (SD)** | 69.5 | (43.9) | 78.2 | (47.2) | 66.5 | (44.9) | 73.6 | (45.2) | 63.9 | | (44.9) | 74.6 | (45.0) | |
| **Weight, kg; mean (SD)** | 71.8 | (12.6) | 68.3 | (10.9)* | 71.7 | (10.5) | 70.4 | (12.5) | 69.4 | | (12.4) | 71.0 | (12.1) | |
| **Height, m; mean (SD)** | 1.6 | (0.2) | 1.6 | (0.2) | 1.6 | (0.2) | 1.6 | (0.2) | 1.6 | (0.2) | | 1.6 | (0.2) | |
| **Total ST, min/d; mean (SD)** | 425.6 | (184.6) | 420.9 | (178.8) | 407.1 | (176.9) | 427.2 | (184.0) | 453.1 | | (186.7) | 414.8 | (180.8) | |
| **Sleep characteristics** |  | | | |  | | | |  | | | | | |
| **Hours of night-time sleep**; % |  | | | |  | | | |  | | | | | |
| Short sleep (≤6 h) | 32.2 | | 30.3 | | 34.1 | | 30.9 | | 32.9 | | | 31.1 | | |
| Normal sleep | 50.1 | | 51.7 | | 48.8 | | 51.1 | | 48.6 | | | 51.2 | | |
| Long sleep (≥9 h) | 16.9 | | 17.5 | | 15.7 | | 17.5 | | 17.7 | | | 17.0 | | |
| **Overall sleep quality; %** |  | | | |  | | | |  | | | | | |
| Very good | 4.8 | | 9.4 | | 4.1 | | 6.9 | | 4.1 | | | 6.9 | | |
| Good | 52.4 | | 57.8 | | 47.9 | | 55.5 | | 49.8 | | | 55.2 | | |
| Fair | 21.5 | | 16.9 | | 21.2 | | 19.8 | | 20.6 | | | 19.9 | | |
| Poor | 4.3 | | 3.6 | | 5.1 | | 3.8 | | 4.5 | | | 3.9 | | |
| Very poor | 1.7 | | 0.6 | | 1.8 | | 1.1 | | 2.1 | | | 1.1 | | |
| **Health-related variables** |  | | | |  | | | |  | | | | | |
| SF-12, PCS | 46.2 | (10.9) | 49.0 | (9.2)* | 45.7 | (11.3) | 47.4 | (10.1) | 43.8 | | (12.9) | 48.0 | (9.5)* | |
| SF-12, MCS | 53.4 | (9.8) | 53.8 | (8.2)* | 53.6 | (10.3) | 53.5 | (9.1) | 52.9 | | (10.6) | 53.6 | (8.9)* | |
| GHQ score | 9.4 | (3.9) | 8.8 | (3.5) | 9.8 | (4.7) | 9.03 | (3.5)* | 10.0 | | (4.6) | 9.0 | (3.5)* | |
|  |  | | | |  | | | |  | | | | | |

| **Supplementary Table 2.** Continued. | | | | | | | | | | | | | | |
| --- | --- | --- | --- | --- | --- | --- | --- | --- | --- | --- | --- | --- | --- | --- |
|  | **Musculoskeletal** | | | | | **Depression** | | | | | **Cancer** | | | |
|  | **With**  (n=665) | | | **Without**  (n=427) | | **With**  (n=172) | | **Without**  (n=917) | | | **With**  (n=111) | | **Without**  (n=980) | |
| **Socio-demographic variables** |  | | |  | |  | |  | | |  | | | |
| **Age, years; mean (SD)** | 81.0 | (5.6) | | 79.3 | (5.5) | 79.9 | (5.6) | 80.4 | | (5.6) | 79.6 | (4.8) | 80.4 | (5.7)* |
| **Female; %** | 78.6 | | | 47.5 | | 83.7 | | 63.2 | | | 57.7 | | 67.4 | |
| **Education; %** |  | | | | |  | | | | |  | | | |
| Illiterate | 17.3 | | | 10.8 | | 22.7 | | 13.1 | | | 11.7 | | 15.1 | |
| Primary | 58.5 | | | 51.8 | | 57.6 | | 55.7 | | | 48.6 | | 56.6 | |
| Secondary | 10.1 | | | 18.3 | | 9.3 | | 14.0 | | | 14.4 | | 13.2 | |
| University | 5.1 | | | 13.1 | | 3.5 | | 9.2 | | | 12.6 | | 7.8 | |
| **Marital status; %** |  | | |  | |  | | | | |  | | | |
| Single | 2.7 | | | 6.1 | | 2.9 | | 4.3 | | | 1.8 | | 4.3 | |
| Married | 52.3 | | | 66.5 | | 51.7 | | 59.1 | | | 58.6 | | 57.9 | |
| Divorced | 2.1 | | | 2.6 | | 1.2 | | 2.5 | | | 1.8 | | 2.3 | |
| Widowed | 42.7 | | | 24.8 | | 44.2 | | 34.0 | | | 37.8 | | 35.4 | |
| **Income**; % |  | | | | |  | | | | |  | | | |
| ≤600€ per month | 26.8 | | | 16.2 | | 26.7 | | 21.8 | | | 15.3 | | 23.4 | |
| >600≤900€ per month | 30.2 | | | 27.6 | | 34.3 | | 28.2 | | | 27.0 | | 29.5 | |
| >900€ per month | 23.6 | | | 44.3 | | 24.4 | | 33.2 | | | 35.1 | | 31.3 | |
| **Living alone; %** | 31.6 | | | 21.5 | | 31.4 | | 26.9 | | | 24.3 | | 28.0 | |
| **Daily socialization; %** | 92.3 | | | 88.5 | | 93.6 | | 90.4 | | | 91.9 | | 90.7 | |
| **Lifestyle-behaviours** |  | | |  | |  | | | | |  | | | |
| **Smokers; %** | 1.2 | | | 4.9 | | 1.7 | | 2.8 | | | 1.8 | | 2.8 | |
| **Alcohol intake; %** |  | | |  | |  | | | | |  | | | |
| Daily | 15.2 | | | 26.2 | | 11.0 | | 21.0 | | | 23.4 | | 19.1 | |
| 3-5 days per week | 3.0 | | | 5.4 | | 2.9 | | 4.1 | | | 3.6 | | 4.0 | |
| 1-2 days per week | 3.2 | | | 3.3 | | 4.1 | | 3.1 | | | 7.2 | | 2.8 | |
| Less than 1 day per week | 10.2 | | | 6.6 | | 12.2 | | 8.2 | | | 9.0 | | 8.8 | |
| Non-drinker | 62.0 | | | 54.3 | | 61.0 | | 58.7 | | | 51.4 | | 59.8 | |
| Stopped recently | 6.5 | | | 4.2 | | 8.7 | | 4.9 | | | 5.4 | | 5.6 | |
| **MEDAS index; mean(SD)** | 6.9 | | (1.7) | 7.2 | (1.8) | 6.8 | (1.8) | 7.1 | (1.8) | | 7.1 | (1.8) | 7.0 | (1.8) |
| **PASE score; mean (SD)** | 67.9 | | (43.6) | 78.9 | (46.9) | 65.2 | (39.7) | 73.6 | (46.0) | | 66.4 | (46.4) | 72.9 | (45.0) |
| **Weight, kg; mean (SD)** | 69.7 | | (12.0) | 72.1 | (12.2) | 68.1 | (11.0) | 71.1 | (12.3) | | 73.4 | (10.7) | 70.3 | (12.3) |
| **Height, m; mean (SD)** | 1.5 | (0.2) | | 1.6 | (0.2) | 1.5 | (0.1) | 1.6 | | (0.2) | 1.6 | (0.9) | 1.6 | (0.2) |
| **Total ST, min/d; mean (SD)** | 413.0 | | (179.0) | 438.9 | (187.5) | 409.5 | (181.0) | 425.8 | (183.0) | | 445.4 | (174.3) | 420.9 | (183.7) |
| **Sleep characteristics** |  | | |  | |  | | | | |  | | | |
| **Hours of night-time sleep**; % |  | | |  | |  | | | | |  | | | |
| Short sleep (≤6 h) | 33.7 | | | 28.1 | | 23.8 | | 33.0 | | | 38.7 | | 30.7 | |
| Normal sleep | 48.3 | | | 54.3 | | 52.3 | | 50.4 | | | 42.3 | | 51.6 | |
| Long sleep (≥9 h) | 17.1 | | | 17.1 | | 23.3 | | 15.8 | | | 18.0 | | 16.9 | |
| **Overall sleep quality**; % |  | | |  | |  | | | | |  | | | |
| Very good | 3.6 | | | 10.5 | | 2.9 | | 7.0 | | | 6.3 | | 6.3 | |
| Good | 50.4 | | | 59.7 | | 46.5 | | 55.4 | | | 48.6 | | 54.7 | |
| Fair | 22.3 | | | 16.6 | | 23.8 | | 19.4 | | | 20.7 | | 19.9 | |
| Poor | 5.1 | | | 2.3 | | 4.7 | | 3.9 | | | 4.5 | | 4.0 | |
| Very poor | 1.8 | | | 0.5 | | 1.7 | | 1.2 | | | 1.8 | | 1.2 | |
| **Health-related variables** |  | | |  | |  | | | | |  | | | |
| SF-12. PCS | 44.5 | | (11.1) | 50.8 | (8.1)* | 43.2 | (11.5) | 47.8 | (10.1)* | | 44.2 | (13.0) | 47.4 | (10.1)* |
| SF-12. MCS | 53.4 | | (9.7) | 53.6 | (8.7)* | 51.1 | (11.3) | 53.9 | (8.8)* | | 54.5 | (10.1) | 53.4 | (9.2) |
| GHQ score | 9.7 | | (4.1) | 8.4 | (3.1)* | 11.1 | (5.2) | 8.9 | (3.4)* | | 9.8 | (4.4) | 9.1 | (3.7) |
|  |  | | | | |  | | | | |  | | | |
| Variables are presented as mean (standard deviation) or as prevalence (%) of participants in that category. *Statistical significance (p-value<0.05) in the paired sample t-test for change between groups. Abbreviations: SD, standard deviation; MEDAS, Mediterranean Diet Assessment Score; PASE, Physical Activity Scale for the Elderly; ST, sedentary time; SF-12, 12-Item Short-Form Health Survey; PCS, Physical Component Score of the SF-12; MCS, Mental Component Score of the SF-12; GHQ, General Health Questionnaire; CVD, Cardiovascular disease. Higher scores in the MCS and PCS of the SF-12, PASE, as well as on the MEDAS, and lower scores in the GHD are indicative of better health. | | | | | | | | | | | | | | |
