# Supplementary material for: The medium-term consequences of a COVID-19 lockdown on lifestyle among Spanish older people with hypertension, pulmonary disease, cardiovascular disease, musculoskeletal disease, depression, and cancer
Source: Epidemiol Health. 2022 Feb 21;44:e2022026. doi: 10.4178/epih.e2022026 (PMC9684008; doi:10.4178/epih.e2022026)
Supplement: Supplementary Material 3. — Socio-demographic, lifestyle, and health-related characteristics of the study population during COVID 19 lockdown comparing the participants who had completed the 2nd assessment and those lost to follow-up. [file epih-44-e2022026-suppl3.docx]

| **Supplementary Material 3.** Socio-demographic, lifestyle, and health-related characteristics of the study population during COVID 19 lockdown comparing the participants who had completed the 2nd assessment and those lost to follow-up. | | | | |
| --- | --- | --- | --- | --- |
|  | **Whole group** | | | |
|  | **Completed** (n=1092) | | **Lost** (n=385) | |
| **Socio-demographic variables** |  | |  | |
| **Age, years**; mean (SD) | 80.3 | (5.6) | 81.2 | (6.3)* |
| **Female**; % | 66.5 | | 65.7 | |
| **Education**; % |  | |  | |
| Illiterate | 14.7 | | 14.5 | |
| Primary | 55.9 | | 51.2 | |
| Secondary | 13.3 | | 7.8 | |
| University | 8.2 | | 7.5 | |
| **Marital status**; % |  | |  | |
| Single | 4.0 | | 4.2 | |
| Married | 57.9 | | 53.5 | |
| Divorced | 2.3 | | 2.9 | |
| Widowed | 35.7 | | 39.5 | |
| **Income**; % |  | |  | |
| ≤600€ per month | 22.6 | | 20.5 | |
| >600≤900€ per month | 29.2 | | 28.1 | |
| >900€ per month | 31.7 | | 26.5 | |
| **Living alone; %** | 27.7 | | 28.1 | |
| **Daily socialization; %** | 90.8 | | 90.4 | |
| **Lifestyle-behaviours** |  | |  | |
| **Smokers; %** | 2.7 | | 2.3 | |
| **Alcohol intake; %** |  | |  | |
| Daily | 19.5 | | 17.1 | |
| 3-5 days per week | 3.9 | | 1.8 | |
| 1-2 days per week | 3.2 | | 2.3 | |
| Less than 1 day per week | 8.8 | | 8.6 | |
| Non-drinker | 59.0 | | 63.4 | |
| Stopped recently | 5.6 | | 6.8 | |
| **MEDAS index; mean (SD)** | 7.0 | (1.8) | 6.9 | (1.7) |
| **PASE score; mean (SD)** | 72.2 | (45.2) | 58.9 | (41.8) |
| **Weight, kg; mean (SD)** | 70.6 | (12.1) | 69.8 | (11.8) |
| **Height, m; mean (SD)** | 1.6 | (0.2) | 1.6 | (0.1) |
| **Total ST, min/d; mean (SD)** | 423.3 | (182.7) | 383.5 | (207.1)* |
| **Sleep characteristics** |  | |  | |
| **Hours of night-time sleep**; % |  | |  | |
| Short sleep (≤6 h) | 31.5 | | 29.6 | |
| Normal sleep | 50.6 | | 46.8 | |
| Long sleep (≥9 h) | 17.1 | | 20.8 | |
| **Overall sleep quality; %** |  | |  | |
| Very good | 6.3 | | 5.5 | |
| Good | 54.0 | | 50.9 | |
| Fair | 20.1 | | 19.2 | |
| Poor | 4.0 | | 4.7 | |
| Very poor | 1.3 | | 0.5 | |
| **Health-related variables** |  | |  | |
| SF-12, PCS | 47.1 | (10.4) | 45.1 | (11.5)* |
| SF-12, MCS | 53.5 | (9.3) | 45.7 | (6.9)* |
| GHQ score | 9.2 | (3.8) | 8.9 | (4.2) |
| Variables are presented as mean (standard deviation) or as prevalence (%) of participants in that category. *Statistical significance (p-value<0.05) in the paired sample t-test for changes between groups. Abbreviations: SD, standard deviation; MEDAS, Mediterranean Diet Assessment Score; PASE, Physical Activity Scale for the Elderly; ST, sedentary time; SF-12, 12-Item Short-Form Health Survey; PCS, Physical Component Score of the SF-12; MCS, Mental Component Score of the SF-12; GHQ, General Health Questionnaire; Higher scores in the MCS and PCS of the SF-12, PASE, as well as on the MEDAS, and lower scores in the GHQ are indicative of better health. | | | | |
